# Advancing Dementia Care Interventions With Technology Solutions

**DOI:** 10.1093/geroni/igab046.010

**Published:** 2021-12-17

**Authors:** Jinmyoung Cho, Elena Fazio

## Abstract

Over the past two decades, a number of interventions have been developed and tested to help meet the complex care needs of persons living with dementia (PLWD) and the family care support system. Despite the large foundation of empirical evidence, they are often not readily available as part of dementia care support services. Interventions leveraging technology-based solutions have the potential to bolster their desirability, efficacy, and feasibility. While progress has been made, there is still a need to design and test new innovative solutions in real-world settings. This symposium will highlight three such innovative technology solutions for dementia care and explore lessons learned in their development and testing. Smith et al. demonstrate the feasibility of using a novel in-situ sensor system to assess daily functions for PLWD in home or assisted care settings. Results of detecting and classifying diverse forms of functional assessment and environmental conditions will be discussed in the presentation. Czaja et al. describe a randomized controlled trial evaluating the feasibility and efficacy of an innovative dyadic intervention (DT) delivered through an interactive technology. Recruitment challenges and lessons learned from the feasibility of implementing a dyadic intervention will be presented. Stevens et al. introduce an online approach to delivering REACH II, GamePlan4Care (GP4C). Qualitative thematic analyses from GP4C user test sessions related to both the content and technical features will be discussed. Discussant Dr. Elena Fazio will address the role of technology solutions as a strategy within dementia care interventions and unique challenges and contributions of each project.

